# The novel H_2_VK-65 clinical risk assessment tool predicts high coronary artery calcium score in symptomatic patients referred for coronary computed tomography angiography

**DOI:** 10.3389/fcvm.2023.1096036

**Published:** 2023-07-03

**Authors:** Yodying Kaolawanich, Natthaporn Prapan, Supamongkol Phoopattana, Thananya Boonyasirinant

**Affiliations:** Division of Cardiology, Department of Medicine, Faculty of Medicine Siriraj Hospital, Mahidol University, Bangkok, Thailand

**Keywords:** coronary artery disease, coronary calcium score, cardiac computed tomographic (CT) imaging, risk prediction, chest pain

## Abstract

**Background:**

Coronary computed tomographic angiography (CCTA) has emerged as a powerful imaging modality for the detection and prognostication of individuals with suspected coronary artery disease (CAD). High amounts of coronary artery calcium (CAC) significantly obscure the interpretation of CCTA. Clinical risk assessment tools and data specific to predictors of high CAC in symptomatic patients are limited.

**Methods:**

Consecutive patients who underwent CAC scan and CCTA to diagnose CAD during 2016–2020 were included. A high CAC score was defined as >400 by Agatston method. Univariate and multivariate analyses were performed to determine the predictors of high CAC. The clinical risk score was derived from factors independently associated with high CAC. The derivation cohort was composed of 465 patients; this score was validated in 98 patients.

**Results:**

The mean age was 63 ± 11 years, 53% were female, and 15.9% had high CAC scores. The independent predictors of high CAC scores were age >65 years (odds ratio [OR] 3.02, 95% confidence interval (95%CI) 1.56–5.85, *p *= 0.001), chronic kidney disease (CKD) (OR 11.09, 95%CI 3.38–36.38, *p *< 0.001), heart failure (OR 6.52, 95%CI 2.23–19.09, *p *= 0.001), hypertension (OR 26.44, 95%CI 9.02–77.44, *p *< 0.001), and vascular diseases, including ischemic stroke/transient ischemic attack and peripheral arterial disease (OR 20.96, 95%CI 4.19–104.86, *p *< 0.001). The H_2_VK-65 (**H**ypertension, **H**eart failure, **V**ascular diseases, C**K**D, and Age > **65**) score allocates 1 point for age >65, 2 points for CKD or heart failure, and 3 points for hypertension or vascular diseases. Using a threshold of ≥4 points, the sensitivity and specificity to detect high CAC was 81% and 80%, respectively. The area under the curve was 0.88 and 0.85 in the derivation and validation cohorts, respectively.

**Conclusion:**

The novel H_2_VK-65 score demonstrated good performance for predicting high CAC scores in symptomatic patients referred for CCTA.

## Introduction

Coronary artery disease (CAD) is currently the leading cause of death worldwide and is predicted to remain so for the next 10 years (1). Coronary computed tomography angiography (CCTA) is being increasingly used to assess symptomatic patients for CAD (2). CCTA detects subclinical coronary atherosclerosis and can also accurately rule out anatomy and functionally significant CAD (3). CCTA also yields prognostic information that can be used to guide preventive therapy in patients with stable chest pain (4).

CCTA is the preferred test for patients with a low-intermediate clinical likelihood of obstructive CAD, no previous diagnosis of CAD, and having characteristics associated with a high likelihood of good image quality. However, despite improved computed tomography (CT) imaging systems and proper preparation, a high burden of coronary artery calcium (CAC) adversely impacts the interpretation of CCTA diagnostic imaging due to the presence of beam hardening artifacts and the partial volume effect (5–8). The prevalence of high CAC (Agatston score >400–1,000) in patients with known or suspected CAD who underwent CCTA was reported to range from 10% to 20% (4, 9, 10), which decreases the accuracy of CCTA for diagnosing obstructive CAD (11–14). Hence, the current European Society of Cardiology guideline does not recommend using CCTA in patients with extensive coronary calcification (2).

The development of a simple clinical risk prediction tool to identify patients at high risk for having high CAC would be useful for identifying patients who may not be suitable for CCTA. Accordingly, the aim of this study was to identify the independent clinical predictors of high CAC in symptomatic patients who underwent clinically indicated CAC scan and CCTA, and to develop a clinical risk assessment tool for predicting high CAC in symptomatic patients.

## Materials and methods

### Study population

Consecutive patients aged ≥18 years who underwent both a CAC scan and CCTA using a dual-source CT scanner for diagnosis of CAD between 2016 and 2020 in a tertiary hospital were included. Patients with a previous history of CAD, including myocardial infarction (MI), percutaneous coronary intervention (PCI), or coronary bypass surgery, were excluded. Patients with incomplete CT examinations were also excluded. The protocol for this study was approved by the institutional review board. The requirement to obtain written informed consent was waived due to the retrospective design of our study.

Clinical symptoms, CAD risk factors, and current medications were obtained from electronic medical records. Chest pain was classified according to the methods published by Diamond and Forrester (15). The pre-test probability of obstructive CAD was calculated using age, gender, and symptoms (2). Hypertension was defined as a self-reported history of hypertension, the use of antihypertensive medication, or a blood pressure of ≥140/90 mmHg. Diabetes was defined as a self-reported history of diabetes and/or receiving anti-diabetic treatment, or a fasting glucose of ≥126 mg/dl. Dyslipidemia was defined as a total cholesterol level of ≥240 mg/dl, a low-density lipoprotein (LDL) cholesterol level of ≥130 mg/dl, a high-density lipoprotein (HDL) cholesterol level of <40 mg/dl, a triglyceride level of ≥200 mg/dl, and/or treatment with a lipid-lowering agent. Vascular diseases included ischemic stroke/transient ischemic attack (TIA) and peripheral arterial disease (PAD). Chronic kidney disease (CKD) was defined as structural or functional kidney damage for ≥3 months with or without decreased estimated glomerular filtration rate (eGFR) (eGFR <60 ml/min/1.73 m^2^) (16). Blood pressure and heart rate data were obtained before CCTA. Laboratory results, including serum creatinine, eGFR, hematocrit, fasting plasma glucose, total cholesterol, HDL cholesterol, LDL cholesterol, and triglyceride, were obtained from the medical records within 3 months of CCTA. The eGFR was calculated using the Chronic Kidney Disease Epidemiology Collaboration (CKD-EPI) equation (17).

### CAC and CCTA protocols

CAC and CCTA scans were performed using a 256-slice scanner (SOMATOM Definition Flash; Siemens Healthcare, Erlangen, Germany) according to established guidelines (18) and institutional protocol at the time of the scan. To reduce the heart rate of patients with a heart rate >65 beats per minute, an oral beta blocker was administrated 1 h before imaging. A 0.3 mg sublingual dose of nitroglycerin was administered before initiation of the CCTA scan. Prior to CCTA, a non-enhanced prospective electrocardiography (ECG)-gated sequential scan was performed to determine CAC scoring using the following parameters: rotation time of 280 ms, slice collimation of 0.6 mm, slice width of 3.0 mm, tube voltage of 120 kV, and tube current of 50 mAs. CCTA was then performed using prospective ECG gating and the following parameters: rotation time of 330 ms and slice collimation of 0.6 mm. The tube voltage and tube current were determined by the scanner software (CARE kV and CARE Dose4D, respectively—both Siemens Healthcare). CCTA acquisition was performed using 50–70 ml of iodinated contrast (iopamidol 370 mg iodine per ml) injected intravenously at 5.0–6.0 ml per second followed by normal saline solution. Automated bolus tracking or timing bolus was used to trigger acquisition.

### Image analysis

Analysis of CAC and CCTA images was performed following the standard protocol on a separate workstation (Syngovia; Siemens Healthineers, Erlangen, Germany). CAC scans were interpreted according to the Agatston method (19), with a high CAC score defined as >400. CCTA images were interpreted in accordance with the Society of Cardiovascular Computed Tomography guidelines (20). Overall CCTA image quality was assessed on a four-point ranking scale, as follows: 4, excellent (no artifacts, unrestricted evaluation); 3, good (minor artifacts, good diagnostic quality); 2, adequate (moderate artifacts, still acceptable and diagnostic); and 1, not assessable (severe artifacts impairing accurate evaluation). Coronary atherosclerotic lesions were quantified for lumen diameter stenosis by visual inspection with two-observer consensus using categories of 0%, 1%–24%, 25%–49%, 50%–69%, 70%–99%, and 100% stenosis or uninterpretable. Obstructive CAD was defined when coronary artery segments exhibited plaque with luminal diameter stenosis ≥70% or ≥50% in the left main (LM) coronary artery. Nonobstructive CAD was defined when coronary artery segments exhibited plaque with luminal diameter stenosis 1%–70% or 1%–50% in the LM coronary artery. Vessels smaller than 2 mm were not evaluated. The extent of CAD was also assessed according to the number of vessels with CAD as 1-vessel, 2-vessel, or 3-vessel/LM disease. Uninterpretable segments were coded as being due to calcification, significant motion artifact, or not well visualized.

### Statistical analysis

All statistical analyses were performed using SPSS Statistics for Windows version 20.0 (SPSS, Inc., Chicago, IL, USA). Continuous variables with normal distribution were presented as mean ± standard deviation, and continuous variables with non-normal distribution were presented as median and interquartile ranges (IQR). The normality of the distribution of variables was examined by the Kolmogorov-Smirnov test. Categorical variables were present as absolute numbers and percentages. Differences between patients with CAC ≤ and >400 in terms of baseline and image characteristics were compared using the Student’s unpaired *t*-test or the Mann-Whitney *U* test for continuous variables, while the chi-square test or Fisher’s exact test was used for categorical variables, as appropriate.

Binary logistic regression analysis was performed to identify significant predictors of high CAC from baseline characteristics. Variables with a *p*-value <0.05 from univariate analysis were entered into multivariate analysis. The results of the univariate and multivariate analyses are given as odds ratios along with their respective 95% confidence intervals. A *p*-value less than 0.05 was considered statistically significant for all tests.

The factors found to be independently associated with high CAC from the multivariate analysis were used to develop a clinical risk assessment tool for predicting high CAC. A risk score was generated using the sum of assigned points, defined as the exponential of the estimated coefficient and rounded to the nearest integer. We performed receiver operating characteristic (ROC) curve analysis to evaluate the diagnostic performance of our score and to determine the best cutoff value for predicting a high CAC score. The accuracy, sensitivity, specificity, positive predictive value (PPV), and negative predictive value (NPV) were calculated. A validation cohort was used to evaluate the accuracy of the developed risk assessment tool.

## Results

### Patient and image characteristics

A total of 487 patients were studied. Twenty were excluded due to having known CAD, while two had incomplete CT examinations, resulting in a final study population of 465 patients in the derivation cohort. The mean age of patients was 63.1 ± 11.3 years, and 53.3% were female. The majority of patients presented to the hospital with atypical angina or dyspnea, and most patients had intermediate CAD pre-test probability (mean: 14.4 ± 9.5%). Seventy-four patients (15.9%) had a high CAC score (>400). Baseline characteristics of patients in the derivation cohort stratified according to CAC scores are shown in Table 1. Patients with high CAC scores were older, had more CAD risk factors, and had a significantly higher pre-test CAD probability than those with lower CAC scores. Patients with high CAC scores also had a significantly higher prevalence of heart failure, vascular diseases, and CKD, and were significantly more likely to be taking antithrombotics, antihypertensives, or statins.

**Table 1:** Baseline characteristics of patients in the derivation cohort stratified according to CAC scores.

Characteristics	Total	CAC ≤400	CAC >400	*p*-value
	(*n* = 465)	(*n* = 391)	(*n* = 74)	
Age (years)	63.1 ± 11.3	61.9 ± 11.1	69.9 ± 9.9	***<0***.***001***
Female gender	248 (53.3%)	205 (52.4%)	43 (58.1%)	0.37
Body mass index (kg/m^2^)	25.6 ± 4.8	25.4 ± 4.2	26.5 ± 7.0	0.08
Systolic BP (mmHg)	134.9 ± 19.7	132.6 ± 18.3	147.3 ± 22.5	***<0***.***001***
Diastolic BP (mmHg)	76.3 ± 12.9	75.7 ± 12.1	79.8 ± 16.1	***0***.***01***
Heart rate (beats per min)	61.6 ± 7.9	61.4 ± 7.8	62.7 ± 9.1	0.21
Pre-test CAD probability (%)	14.4 ± 9.5	13.8 ± 9.1	17.4 ± 10.6	***0***.***002***
Symptoms				
Typical angina	32 (6.9%)	25 (6.4%)	7 (9.5%)	0.34
Atypical angina	149 (32.0%)	124 (31.7%)	25 (33.8%)	0.73
Dyspnea	173 (37.2%)	135 (34.5%)	38 (51.4%)	***0***.***01***
CAD risk factors				
Hypertension	222 (47.7%)	153 (39.1%)	69 (93.2%)	***<0***.***001***
Diabetes mellitus	90 (19.4%)	66 (16.9%)	24 (32.4%)	***0***.***002***
Hyperlipidemia	306 (65.8%)	247 (63.2%)	59 (79.7%)	***0***.***01***
Family history of premature CAD	16 (3.4%)	15 (3.8%)	1 (1.4%)	0.49
Current smoker	32 (6.9%)	25 (6.4%)	7 (9.5%)	0.34
Number of risk factors	1.43 ± 1.00	1.29 ± 0.98	2.16 ± 0.79	***<0***.***001***
Clinical history				
Heart failure	28 (6.0%)	15 (3.8%)	13 (17.6%)	***<0***.***001***
Atrial fibrillation	23 (5.0%)	16 (4.1%)	7 (9.5%)	0.07
Vascular diseases^a^	13 (2.8%)	3 (0.8%)	10 (13.5%)	***<0***.***001***
Chronic lung disease	24 (5.2%)	18 (4.6%)	6 (8.1%)	0.25
Chronic kidney disease	18 (3.9%)	7 (1.8%)	11 (14.9%)	***<0***.***001***
Rheumatic disease	6 (1.3%)	3 (0.8%)	3 (4.1%)	0.05
Medications				
ACEI or ARB	145 (31.2%)	101 (25.8%)	44 (59.5%)	***<0***.***001***
Anticoagulant	13 (2.8%)	8 (2.1%)	5 (6.8%)	***0***.***04***
Aspirin	129 (27.7%)	91 (23.3%)	38 (51.4%)	***<0***.***001***
Beta blocker	175 (37.6%)	134 (34.3%)	41 (55.4%)	***0***.***001***
Calcium channel blocker	109 (23.4%)	71 (18.2%)	38 (51.4%)	***<0***.***001***
Diuretic	23 (5.0%)	13 (3.3%)	10 (13.5%)	***0***.***001***
Insulin	4 (0.9%)	2 (0.5%)	2 (2.7%)	0.12
Oral hypoglycemic agent	61 (13.1%)	43 (11.0%)	18 (24.3%)	***0***.***002***
Statin	226 (48.6%)	170 (43.5%)	56 (75.7%)	***<0***.***001***
Thienopyridine	25 (5.4%)	13 (3.3%)	12 (16.2%)	***<0***.***001***
Laboratory results				
Hematocrit (%)	39.7 ± 4.4	40.0 ± 4.2	38.4 ± 4.9	***0***.***01***
Fasting plasma glucose (mg/dl)	104.4 ± 16.1	103.3 ± 14.6	109.4 ± 21.2	***0***.***004***
Total cholesterol (mg/dl)	173.7 ± 37.6	174.1 ± 37.9	171.7 ± 36.2	0.64
HDL cholesterol (mg/dl)	55.7 ± 15.1	55.9 ± 14.8	54.6 ± 16.8	0.52
LDL cholesterol (mg/dl)	95.4 ± 35.0	96.1 ± 35.3	92.2 ± 33.3	0.41
Triglyceride (mg/dl)	122.0 ± 62.0	118.8 ± 55.4	136.7 ± 85.4	***0***.***03***
Serum creatinine (mg/dl)	0.95 ± 0.80	0.90 ± 0.65	1.21 ± 1.32	***0***.***002***
eGFR (ml/min/1.73 m^2^)	79.4 ± 18.0	81.7 ± 16.6	67.7 ± 20.5	***<0***.***001***

Data expressed as number and percentage or mean plus/minus standard deviation.

ACEI, angiotensin converting enzyme inhibitors; ARB, angiotensin II receptor blockers; BP, blood pressure; CAC, coronary artery calcium; CAD, coronary artery disease; eGFR, estimated glomerular filtration rate; HDL, high-density lipoprotein; LDL, low-density lipoprotein.

^a^ Vascular diseases included ischemic stroke/transient ischemic attack and peripheral arterial disease.

Bold italic indicates statistical significance (*p* < 0.05).

Table 2 shows the CAC and CCTA characteristics for all patients in the derivation cohort and compared between the low and high CAC groups. The mean and median CAC scores were 231.4 ± 515.2 and 37.6 (IQR: 0.8–237.8), respectively. Patients with high CAC scores had significantly lower mean CCTA image quality scores (2.90 ± 0.74 vs. 3.71 ± 0.49, respectively; *p *< 0.001), and 7 (9.4%) patients had uninterpretable images due to dense calcification. Patients with high CAC scores also had a significantly higher prevalence of obstructive CAD and multivessel or left main disease.

**Table 2:** CAC and CCTA characteristics of patients in the derivation cohort.

Characteristics	Total	CAC ≤400	CAC >400	*p*-value
	(*n* = 465)	(*n* = 391)	(*n* = 74)	
CAC score (Agatston)				
Mean	231.4 ± 515.2	70.8 ± 100.2	1,080.3 ± 874.5	***<0***.***001***
Median	37.6 (0.8–237.8)	19.6 (0–109.0)	808.2 (581.6–1,235.9)	***<0***.***001***
CCTA				
Mean image quality score	3.58 ± 0.61	3.71 ± 0.49	2.90 ± 0.74	***<0***.***001***
Normal coronaries	105 (22.6%)	105 (26.9%)	0 (0.0%)	***<0***.***001***
Nonobstructive CAD (1–69%)	276 (59.4%)	240 (61.4%)	36 (48.7%)	***0***.***04***
Obstructive CAD (≥70%)	75 (16.1%)	44 (11.3%)	31 (41.9%)	***<0***.***001***
1-vessel	53 (11.4%)	35 (9.0%)	18 (24.3%)	0.07
2-vessel	8 (1.7%)	3 (0.8%)	5 (6.8%)	***<0***.***001***
3-vessel or left main	14 (3.0%)	6 (1.5%)	8 (10.8%)	***<0***.***001***
Uninterpretable degree of stenosis	9 (1.9%)	2 (0.4%)	7 (9.4%)	***<0***.***001***

Data expressed as number and percentage, mean plus/minus standard deviation, or median and interquartile range.

CAC, coronary artery calcium; CAD, coronary artery disease; CCTA, coronary computed tomography angiography.

Bold italic indicates statistical significance (*p* < 0.05).

### Predictors of high CAC score and development of the H_2_VK-65 tool

Table 3 shows the results of univariate and multivariate logistic regression analysis to identify factors independently associated with high CAC scores. The significant predictors of high CAC identified by univariate analysis were age, dyspnea, hypertension, diabetes mellitus, hyperlipidemia, history of heart failure, vascular diseases, CKD, and rheumatic disease. Subsequent multivariate analysis revealed age, hypertension, history of heart failure, vascular diseases, and CKD as independent predictors of high CAC. The age threshold with the highest AUC using a CAC score >400 as the reference was 65 years (AUC: 0.71, *p *< 0.001).

**Table 3:** Univariate and multivariate logistic regression analysis to identify factors associated with high CAC scores.

Factors	Univariate analysis		Multivariate analysis	
	OR (95%CI)	*p*-value	OR (95%CI)	*p*-value
Age >65 years	3.60 (2.10–6.16)	***<0***.***001***	3.02 (1.56–5.85)	***0.001***
Female gender	1.26 (0.76–2.08)	0.37		*** ***
Body mass index	1.04 (0.99–1.09)	0.09		
Typical angina	1.53 (0.64–3.68)	0.34		
Atypical angina	1.10 (0.65–1.86)	0.73		
Dyspnea	2.00 (1.21–3.30)	***0***.***01***		
Hypertension	21.47 (8.47–54.42)	***<0***.***001***	26.44 (9.02–77.44)	***<0.001***
Diabetes mellitus	2.36 (1.36–4.11)	***0***.***002***		
Hyperlipidemia	2.29 (1.26–4.19)	***0***.***01***		
Family history of premature CAD	0.34 (0.05–2.64)	0.30		
Current smoker	1.53 (0.64–3.68)	0.34		
Heart failure	5.34 (2.42–11.78)	***<0***.***001***	6.52 (2.23–19.09)	***0.001***
Atrial fibrillation	2.45 (0.97–6.18)	0.06		*** ***
Vascular diseases^a^	20.21 (5.41–75.42)	***<0***.***001***	20.96 (4.19–104.86)	***<0.001***
Chronic lung disease	1.83 (0.70–4.77)	0.22		*** ***
Chronic kidney disease	9.58 (3.58–25.63)	***<0***.***001***	11.09 (3.38–36.38)	***<0.001***
Rheumatic disease	5.47 (1.08–27.62)	***0***.***04***		

CAC, coronary artery calcium; CAD, coronary artery disease; CI, confidence interval; OR, odds ratio.

^a^ Vascular diseases included ischemic stroke/transient ischemic attack and peripheral arterial disease.

Bold italic indicates statistical significance (*p* < 0.05).

**Figure 1:**
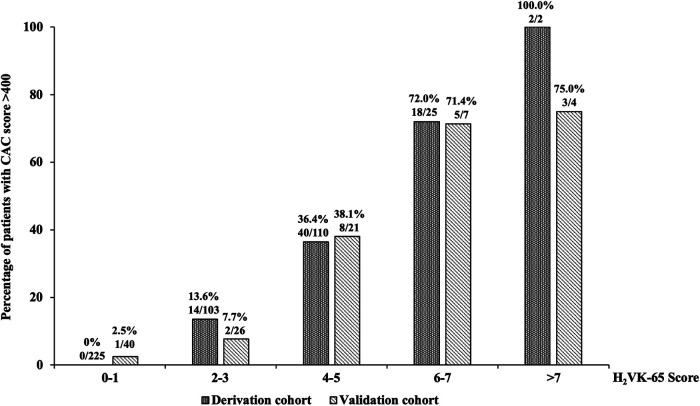
The prevalence of patients with high coronary artery calcium (CAC) scores relative to H_2_VK-65 scoring compared between the derivation and validation cohorts.

We developed a clinical risk assessment tool to predict high CAC using an estimated coefficient (B) for each independent predictor, as follows: age >65 years (B: 1.11); hypertension (B: 3.28); history of heart failure (B: 1.88): vascular disease (B: 3.04); and CKD (B: 2.41). The H_2_VK-65 score (**H**ypertension, **H**eart failure, **V**ascular disease, C**K**D, and Age >**65**), which ranges from 0 to 11, allocates 1 point for age >65 years, 2 points for CKD or heart failure, and 3 points for hypertension or vascular diseases. Figure 1 shows the prevalence of patients with high CAC scores relative to H_2_VK-65 scoring compared between the derivation and validation cohorts. As demonstrated in the figure, the number of patients with high CAC scoring increases as the H_2_VK-65 score increases. No patients in the derivative cohort with an H_2_VK-65 score of 0 or 1 had a CAC score >400 (0 from 225 patients).

**Figure 2:**
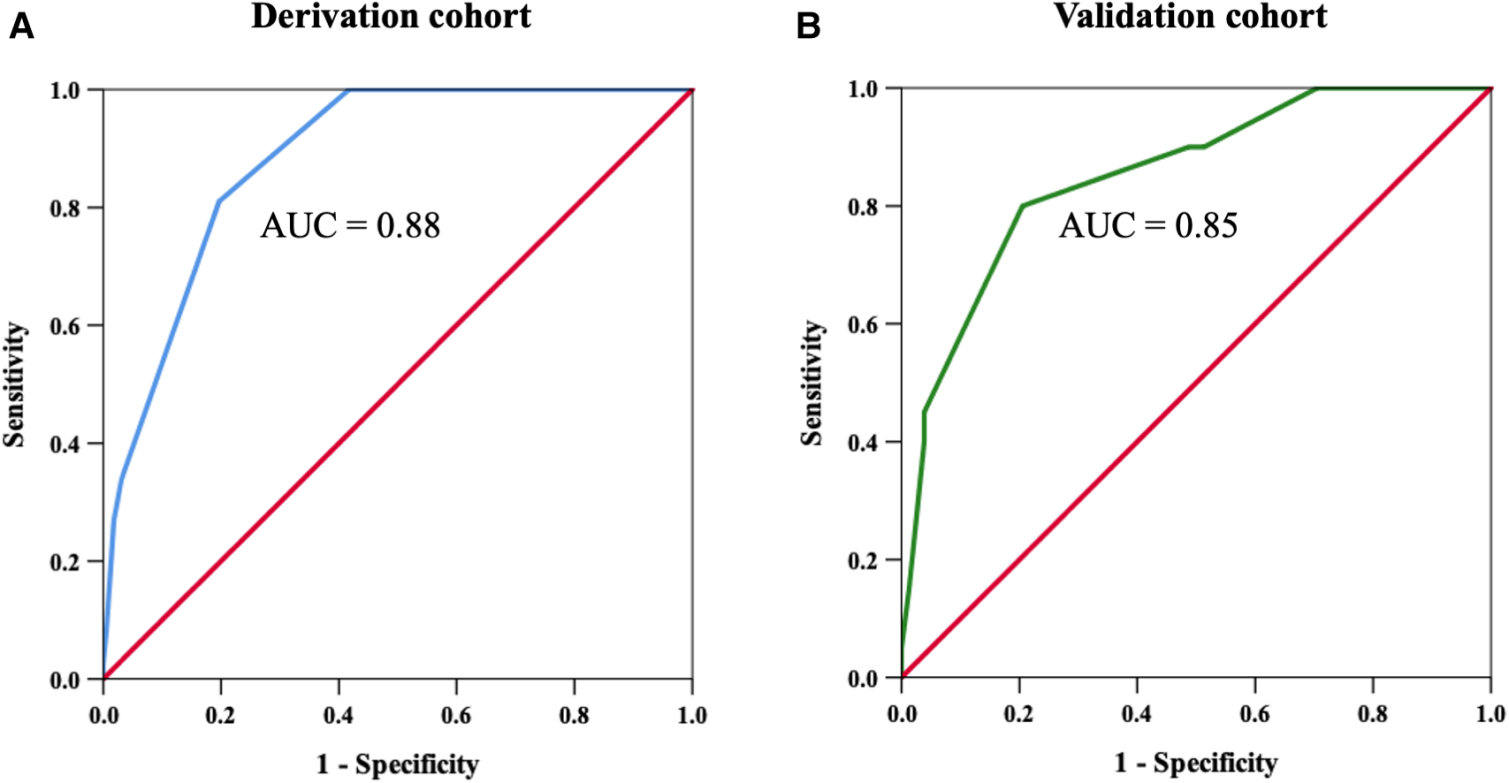
Receiver operating characteristic curves to determine the H_2_VK-65 score cutoff with the highest sensitivity and specificity for predicting high coronary artery calcium (CAC) score in the derivation cohort (**A**), and to confirm the identified cutoff value in the validation cohort (**B**). Using a cutoff threshold of 4 points or above, the sensitivity and specificity for detecting high CAC was 81% and 80%, respectively, in the derivation cohort. The area under the curve was 0.88 and 0.85 in the derivation cohort and validation cohort, respectively.

An H_2_VK-65 cutoff value of ≥4 was shown to have an AUC of 0.88 (95%CI: 0.85–0.92) (Figure 2A and Table 4), and a sensitivity of 0.81 (95%CI: 0.70–0.89), specificity of 0.80 (95%CI: 0.76, 0.84), positive predictive value (PPV) of 0.44 (95%CI: 0.38–0.49), and negative predictive value (NPV) of 0.95 (95%CI: 0.93–0.97) for predicting the presence of high CAC in symptomatic patients (Table 4). Therefore, an H_2_VK-65 score <4 is considered negative for high CAC, and a score ≥4 is considered positive for high CAC in symptomatic patients. Furthermore, we assessed the H_2_VK-65 score with a cutoff of ≥4 to predict a CAC score >1,000. Among the 28 patients with a CAC score >1,000, the AUC was 0.85 (95%CI: 0.80–0.90), sensitivity was 0.89 (95%CI: 0.72–0.98), specificity was 0.74 (95%CI: 0.70–0.78), accuracy was 0.75 (0.71, 0.79), PPV was 0.18 (0.12, 0.26), and NPV was 0.99 (0.97, 0.99).

**Table 4:** Diagnostic performance of the H_2_VK-65 scores for identifying symptomatic patients with high CAC in the derivation and validation cohorts.

Cohort	Prevalence of High CAC score	AUC (95%CI)	Accuracy (95%CI)	Sensitivity (95%CI)	Specificity (95%CI)	PPV (95%CI)	NPV (95%CI)
Derivation cohort	74/465 (15.9%)	0.88 (0.85–0.92)	0.80 (0.77–0.84)	0.81 (0.70–0.89)	0.80 (0.76–0.84)	0.44 (0.38–0.49)	0.95 (0.93–0.97)
Validation cohort	20/98 (20.4%)	0.85 (0.76–0.95)	0.80 (0.70–0.87)	0.80 (0.56–0.94)	0.79 (0.69–0.88)	0.50 (0.38–0.62)	0.94 (0.86–0.97)

AUC, area under the curve; CAC, coronary artery calcium; CI, confidence interval; NPV, negative predictive value; PPV, positive predictive value.

### Validation of the H_2_VK-65 score

The developed H_2_VK-65 clinical assessment tool was validated in a separate and different group of 98 patients (validation cohort) that were referred for both CAC and CCTA to diagnose CAD. Baseline patient characteristics compared between the derivation and validation cohorts are shown in Table 5. Most variables, including CAD risk factors, symptoms, and pre-test CAD probability, were non-significantly different between the validation cohort and derivation cohort. The prevalence of high CAC scores and mean and median CAC scores were also non-significantly different between the two cohorts. The diagnostic performance of the H_2_VK-65 scores for identifying symptomatic patients with high CAC compared between the derivation and validation cohorts is shown in Table 4. The AUC of the H_2_VK-65 score for predicting high CAC in the validation cohort was 0.85 (95%CI: 0.76–0.95) (Figure 2B).

**Table 5:** Baseline patient characteristics compared between the derivation and validation cohorts.

Characteristics	Derivation cohort (*n* = 465)	Validation cohort (*n* = 98)	*p*-value
Mean age (years)	63.1 ± 11.3	62.5 ± 11.5	0.63
Female gender	248 (53.3%)	52 (53.0%)	0.96
Body mass index (kg/m^2^)	25.6 ± 4.8	25.1 ± 3.9	0.33
Systolic BP (mmHg)	134.9 ± 19.7	135.4 ± 19.6	0.82
Diastolic BP (mmHg)	76.3 ± 12.9	76.5 ± 10.9	0.89
Heart rate (beats per min)	61.6 ± 7.9	63.3 ± 7.6	0.05
Pre-test CAD probability (%)	14.4 ± 9.5	15.7 ± 9.9	0.22
Symptoms			
Typical angina	32 (6.9%)	6 (6.1%)	0.77
Atypical angina	149 (32.0%)	38 (38.8%)	0.19
Dyspnea	173 (37.2%)	33 (33.7%)	0.52
CAD risk factors			
Hypertension	222 (47.7%)	55 (56.1%)	0.13
Diabetes mellitus	90 (19.4%)	24 (24.4%)	0.26
Hyperlipidemia	306 (65.8%)	64 (65.3%)	0.92
Family history of premature CAD	16 (3.4%)	3 (3.1%)	0.88
Current smoker	32 (6.9%)	5 (5.1%)	0.51
Number of risk factors	1.43 ± 1.00	1.60 ± 1.11	0.13
Clinical history			
Heart failure	28 (6.0%)	4 (4.1%)	0.46
Atrial fibrillation	23 (5.0%)	3 (3.1%)	0.42
Vascular disease^a^	13 (2.8%)	6 (6.1%)	0.10
Chronic lung disease	24 (5.2%)	9 (9.2%)	0.13
Chronic kidney disease	18 (3.9%)	8 (8.2%)	0.06
Rheumatic disease	6 (1.3%)	1 (1.0%)	0.81
Medications			
ACEI or ARB	145 (31.2%)	41 (41.8%)	***0***.***04***
Anticoagulant	13 (2.8%)	0 (0.0%)	0.09
Aspirin	129 (27.7%)	28 (28.6%)	0.86
Beta blocker	175 (37.6%)	31 (31.6%)	0.26
Calcium channel blocker	109 (23.4%)	25 (25.5%)	0.66
Diuretic	23 (5.0%)	8 (8.2%)	0.21
Insulin	4 (0.9%)	2 (2.0%)	0.34
Oral hypoglycemic agent	61 (13.1%)	18 (18.4%)	0.17
Statin	226 (48.6%)	51 (52.0%)	0.54
Thienopyridine	25 (5.4%)	3 (3.1%)	0.34
Laboratory results			
Hematocrit (%)	39.7 ± 4.4	40.4 ± 4.1	0.15
Fasting plasma glucose (mg/dl)	104.4 ± 16.1	110.2 ± 38.9	***0***.***02***
Total cholesterol (mg/dl)	173.7 ± 37.6	181.0 ± 42.0	0.09
HDL cholesterol (mg/dl)	55.7 ± 15.1	54.0 ± 14.5	0.31
LDL cholesterol (mg/dl)	95.4 ± 35.0	102.5 ± 34.8	0.07
Triglyceride (mg/dl)	122.0 ± 62.0	126.0 ± 66.3	0.57
Serum creatinine (mg/dl)	0.95 ± 0.80	1.04 ± 1.01	0.34
eGFR (ml/min/1.73 m^2^)	79.4 ± 18.0	77.7 ± 19.2	0.40
CAC score (Agatston)			
Mean ± SD	231.4 ± 515.2	236.7 ± 469.9	0.92
Median (IQR)	37.6 (0.9–237.8)	17.9 (0–229.0)	0.09
High CAC score	74 (15.9%)	20 (20.4%)	0.28

Data expressed as number and percentage, mean plus/minus standard deviation, or median and interquartile range.

ACEI, angiotensin converting enzyme inhibitors; ARB, angiotensin II receptor blockers; BP, blood pressure; CAC, coronary artery calcium; CAD, coronary artery disease; eGFR, estimated glomerular filtration rate; HDL, high-density lipoprotein; IQR, interquartile range; LDL, low-density lipoprotein; SD, standard deviation.

^a^ Vascular diseases included ischemic stroke/transient ischemic attack and peripheral arterial disease.

Bold italic indicates statistical significance (*p* < 0.05).

## Discussion

Our results demonstrated that 15.9% of symptomatic patients who underwent CCTA for diagnosis of CAD had a high CAC score (>400 by the Agatston method). Age, hypertension, history of heart failure, vascular diseases, and CKD were identified as independent predictors of high CAC scores. The H_2_VK-65 scoring system is a new and simple clinical assessment score that was designed to predict high CAC with high sensitivity and specificity. The H_2_VK-65 score was also internally validated in another group of patients, and diagnostic performance very similar to that found in the derivation cohort was observed.

CAD is one of the most common types of heart disease in both developing and developed countries, and it was the cause of more than 380,000 deaths globally in 2020 (21). Prevention and early detection of CAD are, therefore, essentially important. CAC is now established as a reliable tool for estimating the risk of MI, coronary death, and all-cause mortality (22, 23). Current guidelines endorse the use of non-contrast CT to assess CAC in suitable asymptomatic patients to improve clinical risk evaluation (23, 24). CCTA is considered a recommended modality in patients with suspected CAD. Given its excellent negative predictive value for excluding CAD in patients with low-intermediate pre-test probability (2), the use of CCTA is now rapidly increasing. However, CCTA is not recommended for patients with extensive CAC due to beam hardening and blooming artifacts caused by dense calcifications (2, 25). Multiple studies have reported the limited specificity, accuracy, and PPV of CCTA in patients with high CAC (7, 8, 11–14). Moreover, during earlier use of CCTA, patients with very high CAC scores (400–1,000) often had their CCTA studies canceled.

The CAC score was previously reported to be an independent predictor of cardiovascular risk compared to conventional epidemiological scores, and an increasing calcium burden was found to be associated with an increased burden of atherosclerosis and stenosis (26). In our study, the prevalence of CAC scoring >400 was 15.9%, which is similar to previously reported rates (4, 9, 10, 27). Patients with high CAC scores had significantly lower image quality, and a higher prevalence of uninterpretable images. Moreover, patients with a high CAC score were more likely to be older, to have a higher pre-test probability of obstructive CAD, and to have multiple CAD risk factors, including hypertension, diabetes mellitus, and dyslipidemia. Patients with high CAC scores also had a higher prevalence of CKD and lower eGFR. These observed characteristics concur with those from previous studies (26, 28–31). Age was reported to be one of the strongest predictors of CAC scoring in both men and women (32). Alison et al. found that asymptomatic patients aged >65 years had CAC scores at least six times higher than those observed in patients aged <45 years (32). CAC scores were reported to increase in patients with high blood pressure, and were also found to predict new-onset hypertension (33). Leening et al. demonstrated clear association between the extent of CAC and the risk of heart failure, independent of overt CAD (34).

Until now, no risk score has been developed to predict high CAC in symptomatic patients referred to undergo CCTA. As previously described, 10%–20% of this population had high CAC scores (including 15.9% in the present study) that significantly adversely affected CCTA image quality. In this study, we developed a clinical scoring system using independent predictors of a high CAC score, including age, hypertension, history of heart failure, vascular diseases, and CKD. The H_2_VK-65 score is a new and easy-to-use clinical score to detect high CAC in symptomatic patients. This score reliably ruled out clinically significant high CAC with an NPV of 95% and 94% in the derivation and validation cohorts, respectively. An ideal screening score should have high sensitivity to avoid false-negative results, but also be specific enough to avoid referral of low-risk patients for costly and time-consuming investigations. The H_2_VK-65 score also demonstrated high sensitivity and specificity (>80% for both); however, the PPV was modest due to the low-intermediate prevalence of obstructive CAD in this population. The H_2_VK-65 score was internally validated in another group of patients, and similar diagnostic performance was observed in both the derivation and validation cohorts.

This study has some mentionable limitations. First, although a widely acknowledged weakness of retrospective studies is their vulnerability to missing or incomplete data, we were careful to include only patients with complete data in this study. Second, all included patients were referred for CCTA, so some selection bias cannot be excluded. Patients with a very low or high likelihood of obstructive CAD are not normally referred for diagnostic testing. Moreover, patients with atrial fibrillation or severe kidney disease may be under-represented because all patients had to be eligible for CCTA. Third, there are multiple definitions of high CAC score, and there is no consensus regarding the most suitable definition. Accordingly, our score may not correspond with or should be considered to be validated for all definitions. Fourth, the validation analysis was performed in our center without external validation, and it lacked data specific to other races or ethnicities. Previous studies found some differences in the prevalence and severity of CAC scores according to race and ethnicity (35, 36), and that CAC predicts clinical outcomes in all race/ethnicity groups, including Asians (37, 38). Finally, this study was conducted in a single center that is a large university-based national tertiary referral center. Therefore, further multicenter study in a much larger and more racially diverse study population is needed to validate our results.

## Conclusion

The newly developed H_2_VK-65 clinical assessment tool demonstrated good performance for predicting high CAC scores in symptomatic patients referred for CCTA. This tool will help to identify symptomatic patients who may not be suitable for CCTA due to the high risk of poor/uninterpretable image quality.

## Data Availability

The raw data supporting the conclusions of this article will be made available by the authors, without undue reservation.
